# Catalytic Ozonation Treatment of Coal Chemical Reverse Osmosis Concentrate: Water Quality Analysis, Parameter Optimization, and Catalyst Deactivation Investigation

**DOI:** 10.3390/toxics12090681

**Published:** 2024-09-20

**Authors:** Yihe Qin, Run Yuan, Shaozhou Wang, Xuewei Zhang, Shaojun Luo, Xuwen He

**Affiliations:** 1 School of Environment and Civil Engineering, Dongguan University of Technology, Dongguan 523808, China; qinyihely@163.com (Y.Q.); qyuanrun@163.com (R.Y.); 2 School of Chemical and Environmental Engineering, China University of Mining & Technology, Beijing 100083, China; wangshaozhou95@163.com (S.W.); ethan-z@foxmail.com (X.Z.); shaojun_luo@163.com (S.L.)

**Keywords:** catalytic ozonation, catalyst deactivation, reverse osmosis concentrate, water quality analysis

## Abstract

Catalytic ozone oxidation, which is characterized by strong oxidizing properties and environmental friendliness, has been widely used in organic wastewater treatments. However, problems such as a low organic pollutant removal efficiency and unstable operation during the catalytic ozone treatment process for wastewater remain. To address these disadvantages, in this study, the treatment efficacy of catalytic ozone oxidation on a coal chemical reverse osmosis concentrate was investigated. The basic water quality indicators of the chemical reverse osmosis concentrate were analyzed. The effects of initial pollutant concentration, pH, ozone concentration, and catalyst concentration on the COD removal rate from the coal chemical reverse osmosis concentrate were explored. Water quality indicators of the chemical reverse osmosis concentrate before and after the catalytic ozone treatment were studied using spectroscopic analysis methods. The RO concentrate demonstrated large water quality fluctuations, and the catalytic ozonation process removed most of the pollutants from the treated wastewater. A possible deactivation mechanism of the ozone catalyst was also proposed. This study provides a theoretical reference and technical support for the long-term, efficient, and stable removal of organic pollutants from coal chemical reverse osmosis concentrate using a catalytic ozone oxidation process in practical engineering applications.

## 1. Introduction

Ozone oxidation technology has been widely used in organic wastewater treatments because of its environmental friendliness and absence of secondary pollution [1,2]. However, the ozone oxidation process within wastewater exhibits disadvantages, such as the incomplete mineralization of organic pollutants and the high toxicity of intermediate byproducts [3,4]. Therefore, researchers often utilize advanced oxidation processes based on ozone to treat wastewater, such as ozone/hydrogen peroxide, catalytic ozone oxidation, ozone/micro-nano bubbles, ozone/sulfuric acid, ozone/ultraviolet, and other wastewater treatment processes [5,6,7,8,9]. Compared to other processes with stringent operating conditions, non-homogeneous ozone catalytic oxidation technology is free from the influence of external conditions such as light, heat, and pressure, and is therefore widely used in wastewater treatment processes [10].

Currently, there are various types and compositions of ozone catalysts, which can generally be divided into several categories: self-supporting catalysts (e.g., FeOOH, CeO_2_, ZnFe_2_O_4_, Fe_3_O_4_, pyrite, and so on), loaded catalysts (e.g., Fe/Al_2_O_3_, Fe/ZSM-5, Fe/ZrO_2_, Fe_3_O_4_/activated carbon, Fe-Mn/bauxite, and so on), and other catalysts (e.g., scrap iron, modified sludge, slag, nickel foam, and so on) [11,12]. Owing to their prospects for engineering applications and catalytic effectiveness, loaded catalysts have received extensive attention from scholars, both domestically and internationally [13,14]. Researchers typically load one or more active metals onto aluminum to enhance the catalytic activity and produce acceptable catalytic results [15,16]. In previous studies, Mn-Fe-Mg-Ce/Al_2_O_3_ was successfully used as an ozone catalyst in the treatment of refinery wastewater, and its COD removal efficiency was twice that of a single ozone oxidation system [17]. Studies showed that compared to a single ozone system for treating coking reverse osmosis concentrate, the catalytic ozone system composed of Mn-Ce/*γ*-Al_2_O_3_ could reduce ΔO_3_/ΔCOD by about 37.6% [18]. In addition, ozone catalysts loaded with active metal components on Al_2_O_3_ also showed high catalytic efficiency in the treatment of difficult to treat wastewaters, such as high salt phenol containing wastewater, high salt petrochemical wastewater, and chemical wastewater [19,20,21].

The stability of a catalyst largely determines whether catalytic ozone oxidation technology can be applied long-term in the engineering field of wastewater treatment [10]. Although research on catalytic ozone oxidation for wastewater treatment is extensive, many studies have focused on the treatment of wastewater and the degradation mechanism of organic substances, with less emphasis placed on the deactivation and regeneration processes of the catalyst [22]. Therefore, further attention to the deactivation mechanism of catalysts was expected to promote the service life of catalysts and improve the stability of processing operations. Studies showed that the deactivation of Fe_2_O_3_/Al_2_O_3_•SiO_2_ catalysts during the catalytic ozonation process of coal chemical wastewater may be caused by carbon deposition in the catalyst’s internal pore structure [23]. Meanwhile, studies showed that the organic mucus layer composed of extracellular polymers could also cause catalyst deactivation in high salt petrochemical wastewater [24]. In addition, inorganic salts such as silicates have also been considered as important reasons for the deactivation of ozone catalysts [25]. The different reasons for catalyst deactivation may be due to differences in water quality and the use of different catalysts. Therefore, it is necessary to continue exploring the reasons for catalyst deactivation in the catalytic ozonation process of typical difficult to treat wastewaters.

For modern coal chemical enterprises located in regions of China with a fragile ecological environment, the industrial wastewater they produce is prohibited from being discharged outside, and they are required to reach a threshold of “zero discharge of wastewater [26]”. Considering policy requirements and the cost of industrial water use, recycling coal chemical wastewater has become the primary choice for modern coal chemical enterprises. To meet the requirements for wastewater reuse, chemical coal wastewater generally must be treated using a reverse osmosis (RO) process. However, a large amount of RO concentrate water is produced during the reverse osmosis treatment of coal chemical wastewater. Notably, owing to the addition of front-end process reagents and the multistage concentration of wastewater, coal chemical RO concentrated water has the characteristics of high conductivity and high organic matter, and it has become a typical difficult to treat wastewater [27]. Compared to other water quality indicators of coal chemical reverse osmosis concentrate, organic pollutants in wastewater have been a matter of widespread concern due to their high toxicity and the difficulty of removing them. Due to the superiority of catalytic ozonation technology, it is often applied to the treatment of organic matter in coal chemical RO concentrated water. The catalytic performance of catalysts largely determines the effectiveness of wastewater treatment. Although there are currently many studies on improving the catalytic performance of catalysts, there is still relatively little research on the reasons for catalyst deactivation in the catalytic ozonation process of coal chemical RO concentrate. Therefore, further investigation into the reasons for catalyst deactivation is of great significance for the stable operation of the processing and efficient treatment of wastewater.

In this study, the efficacy of catalytic ozonation on treating coal chemical RO concentrated water was studied. The basic water quality indicators of the wastewater were analyzed, the parameters for treating the wastewater using the catalytic ozone oxidation process were optimized, and possible reasons for catalyst deactivation were investigated. The aim of this study is to provide a technical reference for the long-term stable application of catalytic ozone oxidation.

## 2. Materials and Methods

### 2.1. Experimental Equipment and Procedures

The wastewater used in this study was obtained from reverse osmosis concentrated water after flocculation in a coal-to-gas factory in northwestern China. The spherical particle ozone catalyst and ozone gas used in this study were provided by the same coal chemical enterprise; the active components of the catalyst mainly consisted of manganese and cerium, and its bulk density was 0.6–0.7 t/m^3^. Unless otherwise specified, the used catalyst mentioned in this study was used in the actual engineering of the catalytic ozonation process for about 4 months. All of the experimental reagents used in this study were purchased from Aladdin Company, Shanghai, China, and were of analytical purity.

### 2.2. Experimental Materials

**Figure 1:**
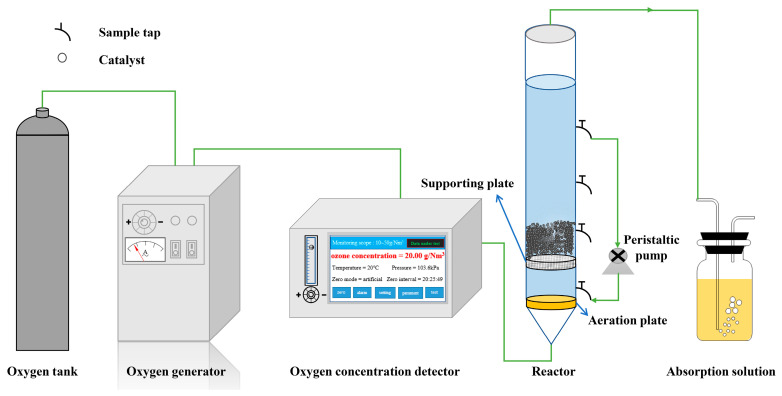
Schematic diagram of the experimental process.

In the ozone catalytic oxidation experiment (Figure 1), the inner diameter and height of the cylindrical high-boron silicon glass reactor were 70 mm and 1.2 m, respectively. The reaction equipment used in the experiment includes oxygen cylinders, ozone generators, ozone concentration detectors, fixed bed reactors, and exhaust gas treatment devices. The wastewater treatment method was the sequential batch method. In addition, to promote the degradation of organic matter in wastewater, the wastewater in the reactor was circulated through a pump for treatment. From the parameter optimization, the effects of different initial COD concentrations (I_1_ = 200 mg/L, I_2_ = 300 mg/L, I_3_ = 400 mg/L, I_4_ = 500 mg/L, I_5_ = 600 mg/L), ozone concentrations (5 mg/L, 10 mg/L, 15 mg/L, 20 mg/L, and 25 mg/L), pH values (4.5, 6, 7.5, 9, and 10.5), and catalyst concentrations (100, 150, 200, 250, and 300 g/L) on the wastewater treatment efficacy were investigated. Unless otherwise specified, the wastewater treatment method was intermittent, with a treatment volume of 1.5 L, ozone dose of 15 mg/L, catalyst concentration of 200 g/L, pH of 7.5, and gas flow rate of 1 L/min. Samples were taken at reaction times of 10, 20, 30, 40, 50, and 60 min.

### 2.3. Analytical Methods

The gaseous ozone concentration at the reactor inlet was measured using an ozone concentration detection instrument. The calculation method for the wastewater COD removal rate is shown in Equation (1). The calculation method for the ozone utilization rate is shown in Equation (1).

(1) $$\sigma =\left(1-\frac{{COD}_{t}}{{COD}_{0}}\right)\times 100$$

An infrared spectroscopy analysis was conducted using a Nicolet 6700 infrared spectrometer (Thermo Fisher Scientific, Cambridge, MA, USA), with a range of 400–4000 cm^−1^. The microscopic characteristics of the catalyst’s surface were explored using X-ray photoelectron spectroscopy (XPS, Thermo Scientific KAlpha). The vacuum degree in the XPS analysis room was 5 × 10^−9^ mbar. The X-ray source was a monochromatic Al Kα source, with an energy of 1486.6 eV, a voltage of 15 kV, and a beam current of 15 mA, and the analysis scanner mode was CAE.

The crystalline characteristics of the catalysts were analyzed using XRD (Bruker D8 ADVANCE, Billerica, MA, USA). The samples were analyzed within the 2θ range of 5–90° with a scanning rate of 5°/min. The XRD data were analyzed using the X’pert HighScore software package 3.0 and standard PDF-2004 cards JCPDS: 44–1481, 02–0629, and 44–0825.

During the analysis process of the three-dimensional fluorescence, a 150 W xenon lamp was used as the light source, with a scanning range of 200–450 nm for the excitation scanning wavelength Ex and 250–550 nm for the emission scanning wavelength Em. The slit widths of the Ex and Em were 5 nm, the scanning interval of the Ex and Em wavelengths was 5 nm, and the scanning speed was 12,000 nm/min. The three-dimensional fluorescence spectrum was divided into five regions. Fluorescence region I (Ex/Em = 200–250 nm/280–330 nm) was related to the aromatic protein I class and tyrosine-like substances. Fluorescence region II (Ex/Em = 200–250 nm/330–380 nm) was related to aromatic protein class II substances, bicyclic aromatic compounds, and heterocyclic compounds [28,29]. Fluorescence regions III (Ex/Em = 200–250 nm/380–550 nm) and IV (Ex/Em = 250–450 nm/280–380 nm) were related to fulvic acid-like substances, humic acid-like substances, microbial products, and polycyclic aromatic hydrocarbons. Fluorescence region V (Ex/Em = 250–450 nm/380–550 nm) was related to humic acid-like substances [30,31].

The detection method used to measure the wastewater quality indicators refers to the fourth edition of the “Water and Wastewater Monitoring and Analysis Methods”. The data acquisition accuracy of the wastewater UV-Vis light spectrum scanning data was 1 nm. The UV indices UV_254_, UV_280_, and UV_355_ and the integral value of the UV spectrum A_190–900_ characterized the degree of aromaticity of organic pollutants in wastewater, the relative changes in protein-like substances, humic acid-like substances, and the total amount of pollutants, respectively [32,33,34].

The elemental composition of the catalyst was determined by X-ray fluorescence spectroscopy (XRF; PANalytical Axios, The Netherlands). The surface morphology of the catalysts was analyzed via scanning electron microscopy (SEM) and energy dispersive X-ray spectroscopy (EDS) using a Zeiss (Germany) Gemini SEM 300 instrument. The structural characteristics of the catalyst were studied using the nitrogen adsorption-desorption isotherm BET method with a Micromeritics (USA) ASAP 2460. The thermal gravimetric TG and differential scanning calorimetry DSC analyses were conducted using a Netzsch (Germany) thermal analysis STA449F3 instrument, and the tests were performed in a nitrogen environment, with a temperature range from room temperature to 1000 °C and a heating rate of 10 °C/min.

The reaction kinetics for the removal of organic pollutants from the coke biochemical effluent using the ozone catalytic oxidation process can be represented by Equation (2):

(2) $$\frac{d{\;[COD]}_{t}}{dt}=\theta_{A}\times \left[O_{3}\right]\times {\left[COD\right]}_{t}^{n}+\theta_{B}\times \left[\cdot OH\right]\times {\left[COD\right]}_{t}^{n}$$

(3) $${\left[COD\right]}_{t}^{-1}-{\left[COD\right]}_{0}^{-1}=\lambda_{2}\times t+C$$

In Equation (2), θ_A_ and θ_B_ are the kinetic reaction constants for the degradation of COD by ozone and ·OH, respectively, and n is the reaction order of COD degradation kinetics, which can be transformed into Equation (3). When *n* = 1 or 2, the reaction kinetics followed pseudo-first-order and pseudo-second-order kinetics, respectively. When *n* = 1 or 2, Equations (2) and (3) can be transformed into Equations (4) and (5), as follows:

(4) $$Ln\frac{{\;[COD]}_{t}}{{\;[COD]}_{0}}={-\lambda }_{1}\times t+C$$

(5) $${\left[COD\right]}_{t}^{-1}-{\left[COD\right]}_{0}^{-1}=\lambda_{2}\times t+C$$

In Equations (4) and (5), λ_1_ and λ_2_ represent the reaction kinetics of pseudo-first-order and pseudo-second-order, respectively, and C is a constant.

## 3. Results

### 3.1. Conventional Wastewater Quality Analysis

Table 1 shows the main water quality indicators of the RO concentrate water used in the experiment. The wastewater indicators, such as electrical conductivity, chloride, sulfate, bromide, fluoride, and nitrate, were relatively high. In addition, the ranges of the wastewater pH, alkalinity, calcium hardness, total hardness, color, suspended solids, and turbidity were 6.59, 3000–6000 mg/L, 100–330 mg/L, 300–1000 mg/L, 1985.4–2183.6°, 32–133 mg/L, and 16–77 NTU, respectively. Notably, the COD concentration of the RO concentrate water in this study ranged from 200 to 600 mg/L, with a high degree of water quality fluctuation.

### 3.2. Parameter Optimization

**Figure 2:**
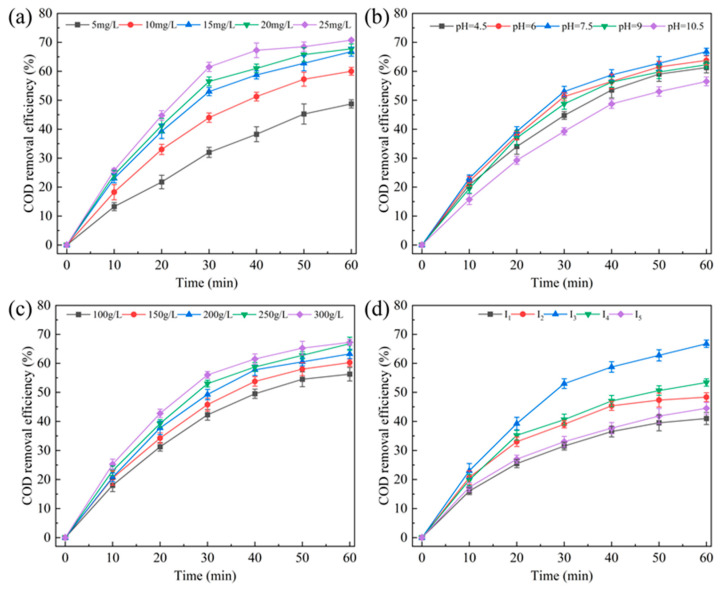
Parameter optimization of the catalytic ozone oxidation process for treating reverse osmosis concentrate water: (**a**) ozone concentration, (**b**) pH, (**c**) catalyst concentration, (**d**) initial pollutant concentration.

In this study, the influence of different ozone concentrations on the efficiency of wastewater COD removal was investigated. When the reaction time was 60 min, the wastewater COD removal efficiencies were 48.71% (5 mg/L), 60% (10 mg/L), 66.75% (15 mg/L), 67.69% (20 mg/L) and 70.74% (25 mg/L) (Figure 2a). The increase in the wastewater removal efficiency may have resulted from the increased production of oxidizing agents in the reaction system [35,36]. Notably, when the ozone concentration was higher than 15 mg/L, the final removal efficiency of wastewater COD did not show a significant increase. Therefore, considering operating costs and energy consumption, an optimal ozone concentration of 15 mg/L was selected.

The pH of wastewater can indirectly affect the removal efficiency of organic pollutants by influencing the catalytic performance [37]. Therefore, in this study, wastewater COD removal efficiency at different pH values was investigated. The removal efficiencies of wastewater COD at different pH values are as follows: pH = 7.50 (66.75%) > pH = 6.00 (63.75%) > pH = 9.00 (62.25%) > pH = 4.50 (61.25%) > pH = 10.50 (56.5%) (Figure 2b). The removal efficiency of wastewater COD was highest when the pH value was 7.50.

Figure 2c shows the influence of different catalyst concentrations (100–300 g/L) on the removal efficiency of organic pollutants from reverse osmosis concentrate water. When the wastewater treatment time was 60 min, the COD removal efficiencies of the coke biochemical effluent at different catalyst concentrations were 300 g/L (67.30%), 250 g/L (66.75%), 200 g/L (63.25%), 150 g/L (60.25%), and 100 g/L (56.20%). The resulting optimal catalyst concentration was 250 g/L.

The efficacy of catalytic ozone oxidation treatment on the wastewater COD under different initial COD concentrations was investigated. The removal efficiencies of organic pollutants at different initial COD concentrations were as follows: I_3_ = 400 mg/L (66.75%), I_4_ = 500 mg/L (53.40%), I_2_ = 300 mg/L (48.33%), I_5_ = 600 mg/L (44.51%), and I_1_ = 200 mg/L (41.00%) (Figure 2d). These results show that when the initial organic matter concentration of the wastewater was 400 mg/L, the removal efficiency of wastewater COD was the highest [38].

### 3.3. Kinetic Analysis

A reaction kinetics study of the COD degradation process in reverse osmosis effluent under different parameters was conducted. The most accurate organic pollutant degradation kinetics model was selected based on determination coefficient (R^2^) results. Table 2, Table 3, Table 4 and Table 5 summarize the pseudo-first-order and pseudo-second-order kinetic models for organic pollutant degradation in coke biochemical effluents under different parameters. The fitting accuracy of the pseudo-second-order reaction kinetic model was more suitable than that of the pseudo-first-order reaction kinetic model.

### 3.4. Spectroscopic Analysis of Wastewater before and after Treatment

#### 3.4.1. Three-Dimensional Fluorescence Analysis

**Figure 3:**
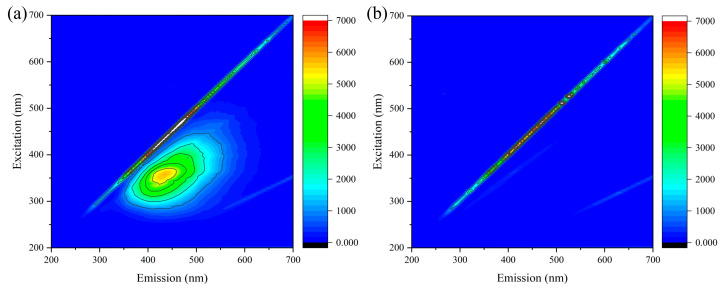
Three-dimensional fluorescence spectra of the reverse osmosis concentrate water (**a**) before and (**b**) after treatment.

Figure 3a shows that the reverse osmosis concentrate water had fluorescence response values in all five fluorescence regions, indicating the presence of aromatic protein-like organic substances, humic acid-like organic substances, fulvic acid-like organic substances, humic acid-like substances, and soluble microbial metabolites in the wastewater [33]. However, the wastewater showed a relatively high concentration of humic acid-like substances and soluble microbial metabolic byproducts in the reverse osmosis concentrate. In addition, Figure 3b shows that the response values of all fluorescence regions of the treated wastewater were substantially reduced [39].

#### 3.4.2. Ultraviolet Spectroscopic Analysis

**Figure 4:**
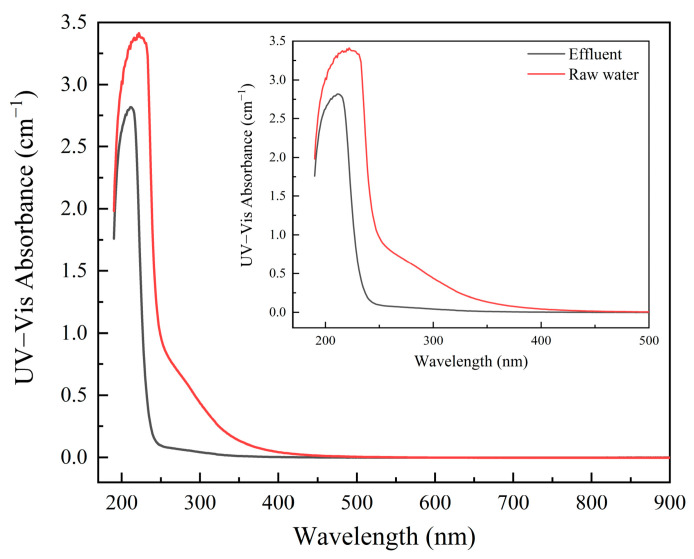
Ultraviolet spectroscopic analysis of wastewater before and after treatment.

Figure 4 shows that the absorption peak of the ultraviolet spectrum of the effluent after catalytic ozone oxidation treatment was substantially reduced. In addition, the changes in the removal rates of the UV index during the catalytic ozone oxidation of the reverse osmosis concentrate water were investigated, and the results showed that the removal efficiencies of UV_254_, UV_280_, UV_355_, and A_190–900_ in the wastewater were 90.24, 90.21, 91.67, and 53.52%, respectively.

#### 3.4.3. FTIR Analysis

**Figure 5:**
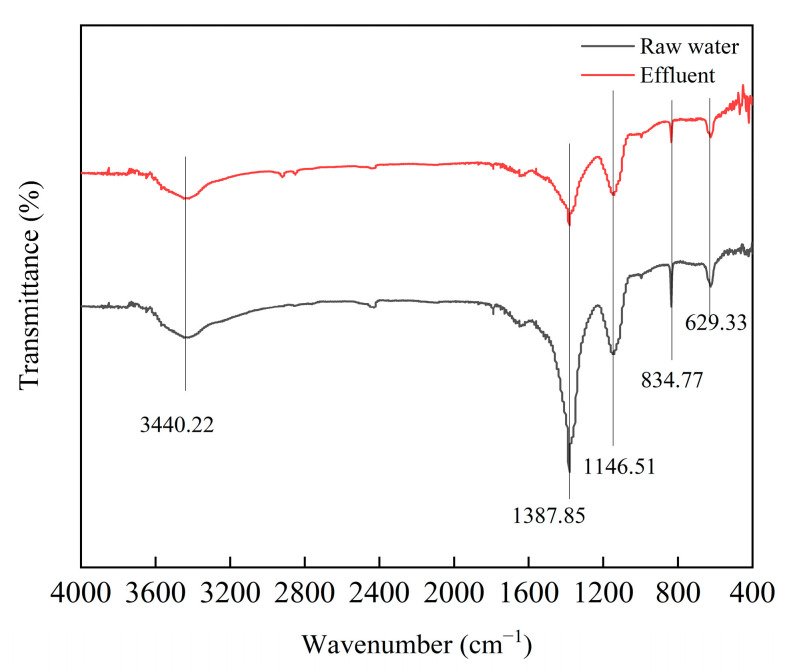
Infrared spectroscopic analysis of wastewater before and after treatment.

FTIR spectroscopy can be used to identify the characteristic functional groups and molecular structures of organic pollutants in wastewater. To analyze the changes in the functional groups of the organic pollutants in the reverse osmosis concentrate water before and after treatment, FTIR studies were conducted (Figure 5). The broad absorption band at −3400–3600 cm^−1^ resulted from the stretching vibration of -OH in phenols, alcohols, and carboxylic acids in water, as well as the stretching vibration of the N-H bond in the -NH_2_ group [40]. The absorption band near 1637.5 cm^−1^ is typically generated by the stretching vibration of the C=O bond in the amide group of water, the C=C vibration in aromatic hydrocarbons, and the C-O vibration in quinones [41]. The peak near 1387.85 cm^−1^ can be attributed to the framework vibration of the C=C bond in aromatic hydrocarbons and the stretching vibration of the C-N bond in amines [42]. The peak at 1146.51 cm^−1^ resulted from the stretching vibration of the C-O bond in esters [43]. The peaks in the 650–900 cm^−1^ range are associated with the out-of-plane bending vibration of the -CH group in aromatic compounds [44]. The peak near 629.33 cm^−1^ can be attributed to the vibration of the C-C=O functional group in aldehydes and ketones or the stretching vibration of the C-Cl bond in halocarbons. The relative intensity differences in the infrared response peaks indicate changes in the molecular structure of the wastewater organic pollutants. As shown in Figure 5, compared with the peak values in the influent, the FTIR peak values in the effluent decreased overall.

### 3.5. Analysis of Catalyst Deactivation Causes

#### 3.5.1. Analysis of Catalytic Performance

**Figure 6:**
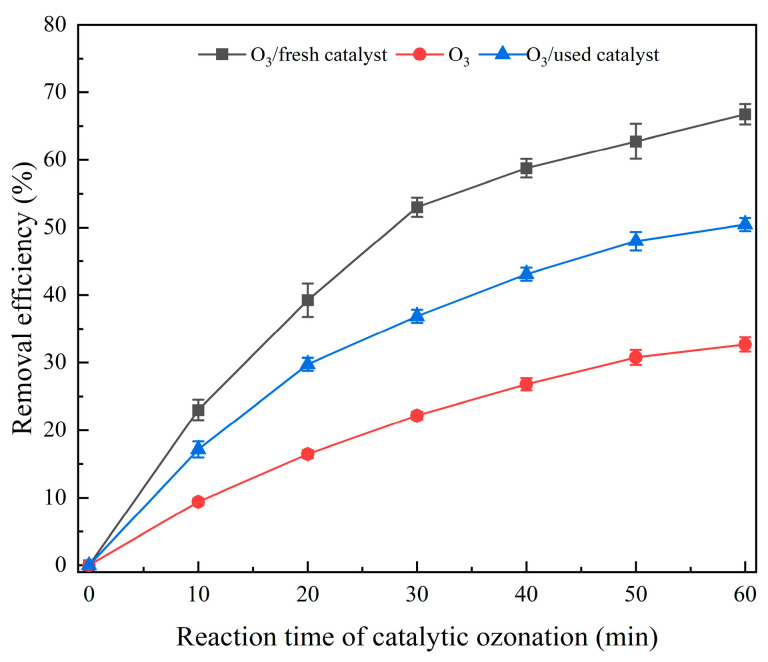
The removal efficiency of COD in RO wastewater before and after using the catalyst.

Figure 6 shows the influence of the fresh and used catalysts on the removal efficiency of organic matter in the RO concentrate water. The COD removal efficiencies in the RO concentrate water for the ozone catalytic oxidation system using a fresh catalyst, the catalytic ozone oxidation system incorporating the used catalyst, and the single ozone system were 66.75, 50.46, and 32.67%, respectively. The results showed that the performance of the fresh catalyst was significantly superior to that of the used catalyst. Compared to the fresh catalyst, the removal efficiency of the used catalyst for wastewater COD decreased by 16.29%.

#### 3.5.2. Analysis of Microscopic Characteristics of Ozone Catalyst before and after Deactivation

##### SEM-EDS Analysis

**Figure 7:**
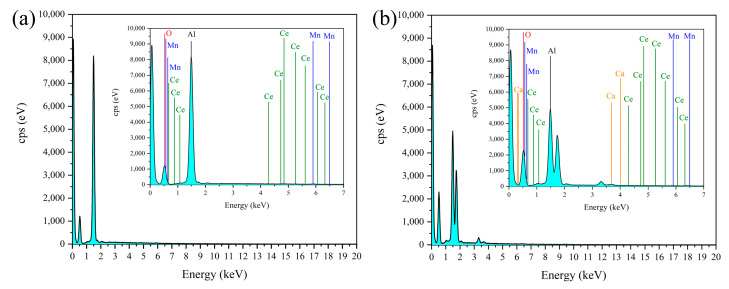
EDS images of (**a**) fresh catalyst and (**b**) used catalyst.

Scanning electron microscopy (SEM-EDS) was used to analyze the morphology and surface components of the catalysts before and after use. Figure 7 and Table 6 show the results of the energy dispersive X-ray spectroscopy EDS analysis of the catalyst before and after use. The elemental composition of the fresh catalyst surface is mainly composed of carrier components, such as oxygen and aluminum, and catalytically active components, such as manganese and cerium. However, the elemental composition of the surface of the used catalyst was mainly carbon, oxygen, aluminum, and calcium; no manganese or cerium was detected.

**Figure 8:**
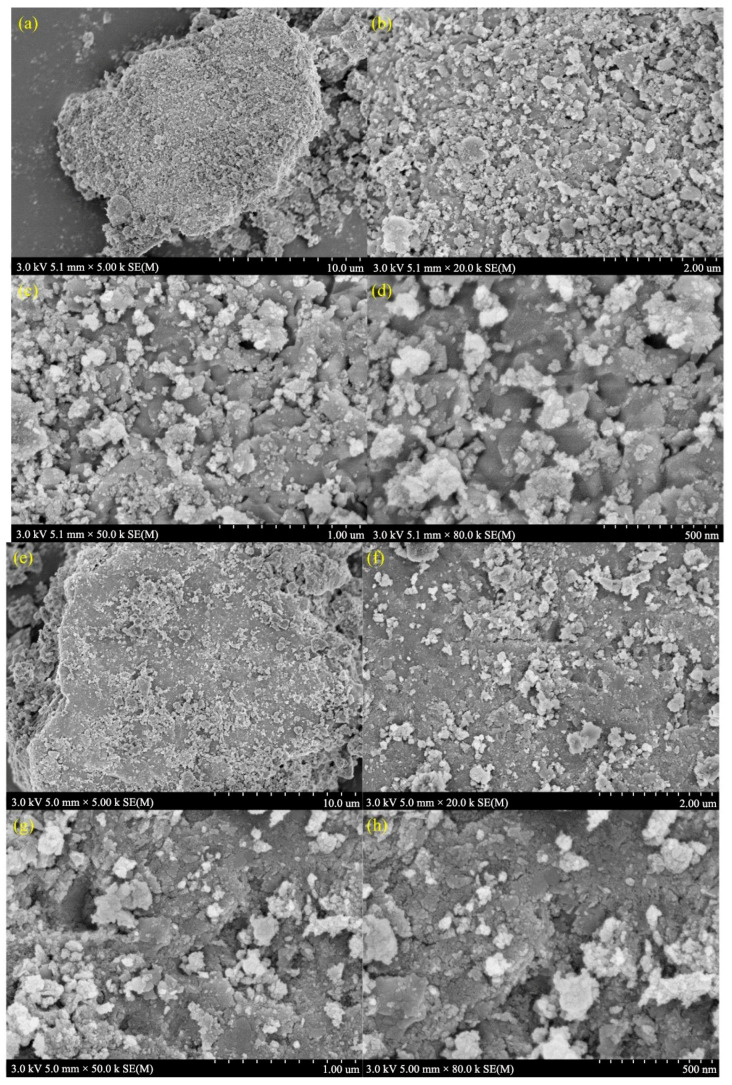
SEM images of the fresh catalyst at magnifications of (**a**) 5000×, (**b**) 20,000×, (**c**) 50,000×, (**d**) 80,000×, and SEM images of the used catalyst at magnifications of (**e**) 5000×, (**f**) 20,000×, (**g**) 50,000×, (**h**) 80,000×.

Figure 8a–d shows the scanning electron microscope images of the fresh catalyst at different magnifications, and Figure 8e–h shows SEM images of the used catalyst at different magnifications. The results showed that the catalyst had a loose and porous structure before use, and that the distribution of the surface substances was relatively uniform. However, the catalyst surface became relatively compact after use, and the distribution of surface substances became chaotic with particle agglomeration.

##### BET Analysis

**Figure 9:**
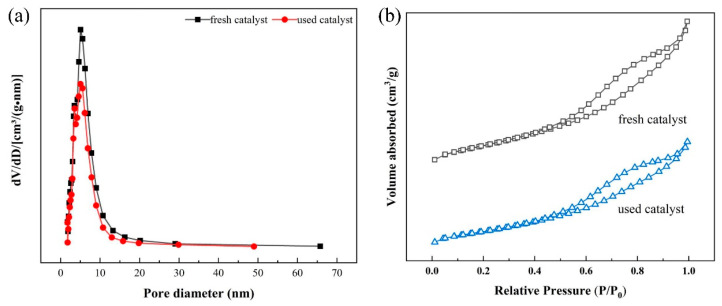
The (**a**) pore size distribution and (**b**) adsorption-desorption isotherm curves of the catalyst before and after use.

Figure 9 shows the nitrogen adsorption-desorption isotherms and pore size distribution of the catalyst before and after use. The fresh catalyst has a BET specific surface area, total pore volume, and average pore diameter of 218.65 m^2^/g, 0.37 cm^3^/g, and 7.45 nm, respectively. The nitrogen adsorption-desorption isotherm of the catalyst exhibited a type-IV adsorption, and the presence of hysteresis loops indicated that the catalyst had a mesoporous structure, which was not demonstrably affected after use. However, compared to the fresh catalyst, the BET specific surface area, total pore volume, and average pore diameter of the used catalyst all showed a decreasing trend, at 162.57 m^2^/g, 0.27 cm^3^/g, and 7.12 nm, respectively.

##### XRD Analysis

**Figure 10:**
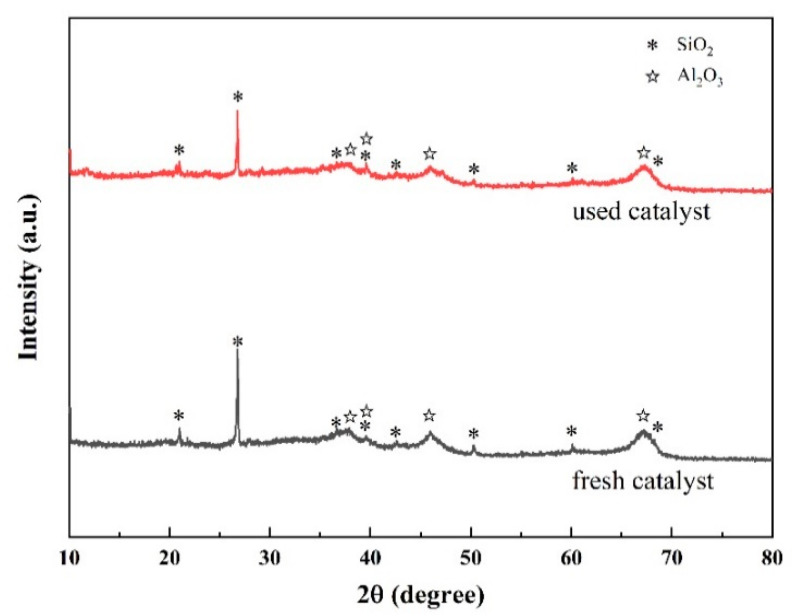
XRD images of the catalyst before and after use.

The XRD analysis of the catalyst before and after use revealed the following results (Figure 10). The diffraction peaks of the fresh catalyst match those of quartz (PDF: 46–1045) and aluminum oxide (PDF: 04–0880). The diffraction peaks at 2θ values of 20.9, 26.6, 36.5, 39.5, 45.8, 50.1, 60, and 67.7° corresponded to the (100), (101), (110), (102), (201), (112), (211), and (212) planes of quartz, respectively. The diffraction peaks at 2θ values of 37.4, 39.7, 45.8, and 67.3° were attributed to the (311), (222), (400), and (441) planes of aluminum oxide, respectively, indicating that the main crystalline phases of the catalyst were silica and aluminum oxide. The crystal phases of the used catalysts did not show significant changes.

##### TG Analysis

To further analyze the surface deposits causing deactivation of the ozone catalyst, the properties of the catalyst before and after use were investigated using thermogravimetric analysis (TGA). The thermogravimetric curves of the catalyst before and after use exhibited a single weight loss process (Figure S1). As the temperature increased from room temperature to 1100 C, the total loss of the fresh catalyst was 19.72%, whereas that of the used catalyst was 11.27%. The difference in the weight loss between the catalysts before and after use was 8.45%.

##### XRF Analysis

To further investigate the main components causing catalyst deactivation, an elemental analysis of the catalyst before and after use was conducted using X-ray fluorescence spectrometry (XRF). Table 7 shows the changes in the elemental compositions of the catalysts before and after use. The results indicate that aluminum oxide and silicon oxide were the main components of the fresh catalyst; their relative contents in the fresh catalyst were approximately 97%, which is consistent with the XRD analysis results. Fresh catalysts included manganese, cerium, sodium, potassium, magnesium, calcium, and sulfur oxides, as well as chlorine. The manganese and cerium oxides comprised the main catalytically active components, whereas the others may represent impurities of the catalyst.

##### XPS Analysis

**Figure 11:**
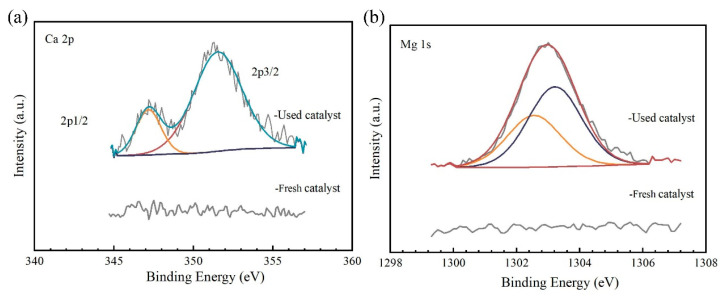
XPS peak analysis of (**a**) Ca and (**b**) Mg before and after catalyst use.

XPS was used to investigate the changes in the elemental composition of the catalysts before and after use. Figure S2 shows the changes in the intensity of the XPS full-spectrum absorption peaks before and after the catalyst was used, indicating an increase in the response peaks related to calcium and magnesium at binding energies of approximately 340 and 1300 eV for the used catalyst [45]. A comparison of the Ca XPS absorption peak intensities before and after the catalyst use is shown in Figure 11a. Compared to the fresh catalyst, the used catalyst exhibited characteristic peaks of Ca(2p_3/2_) and Ca(2p_1/2_) at 351.5 eV and 347.1 eV, respectively; this result may be attributed to the presence of Ca(II) in the sample [46]. Furthermore, Figure 11b indicates the presence of response peaks at binding energies of 1302.6 and 1303.3 eV in the Mg1s spectrum of the used catalyst; this result may have occurred due to the presence of magnesium hydroxide and magnesium oxide in the sample [47].

## 4. Discussion

### 4.1. Water Quality Analysis of RO Concentrate before and after Catalytic Ozonation Process Treatment

Analyzing changes in conventional water quality indicators is beneficial for improving the COD degradation efficiency of wastewater treatment [48]. The results of the water quality analysis showed that the RO concentrate water used in this study had large fluctuations in water quality, a complex composition, high organic pollutant and salt contents, a high hardness, and high suspended solids. Otherwise, the wastewater indicators were relatively high, which may have resulted from an addition of drugs to the front-end process and the concentration of pollutants. According to previous reports, the concentration of organic pollutants in some coal chemical RO concentrated water was relatively low, with COD concentrations ranging from 35 mg/L to 101 mg/L, which differed from the initial water quality of the wastewater used in this study [49,50]. This may be due to differences in the source nodes of coal chemical wastewater. Some studies also showed that the initial COD concentration of the coal chemical RO concentrate was about 835 mg/L, similar to the initial COD concentration of the wastewater in this study [51]. However, it is worth noting that since the wastewater had already undergone reverse osmosis treatment, the water quality indicators such as conductivity and total dissolved solids used in this study were basically consistent with other studies. The above results also indirectly reflect the complexity and difficulty of treating coal chemical RO concentrate.

The catalytic ozonation process could achieve a COD removal efficiency of up to 66.75% for coal chemical RO concentrate, indicating that the organic pollutants in the treated RO concentrate were effectively removed. Moreover, in this paper, spectroscopic analysis methods were also employed to investigate the changes in organic pollutants. The results of Three-Dimensional Fluorescence Analysis indicate that the catalytic ozone oxidation process considerably degraded fluorescent organic substances in the RO concentrate. Some studies showed that advanced oxidation processes based on ozone were effective in degrading large molecular fluorescent organic substances in wastewater, and the three-dimensional fluorescence results of the wastewater in this study are consistent with previous research findings [52,53]. The variation of specific peaks in the UV spectra could represent the changes in corresponding organic compounds in wastewater. In this article, the UV spectral peak of the wastewater treated by catalytic ozonation process showed a significant decrease, which may represent a significant removal of organic matter in the wastewater, consistent with our previous research [33]. In addition, the UV spectrum of the treated wastewater still had a high absorption peak in the short wavelength range, which could be due to the presence of inorganic ions in the wastewater [38]. In the infrared spectrum, the changes at 3440.22 cm^−1^, 1387.85 cm^−1^, 1146.51 cm^−1^, 834.77 cm^−1^, and 629.33 cm^−1^ were most noticeable, indicating that the relevant pollutants in the reverse osmosis concentrate water were effectively removed. Notably, some new peaks appeared in the treated wastewater, which may have resulted from the formation of small-molecule organic pollutants [33,39,54]. This section indicated that the wastewater used in this study was a representative and typical difficult to treat industrial wastewater, which had certain research significance. In addition, as reported by other studies, the catalytic ozonation process in this study could also efficiently remove relevant organic pollutants from wastewater [55,56].

### 4.2. Parameter Optimization and Kinetic Analysis

Appropriate parameter settings could improve the efficiency of the reaction. The removal efficiency of wastewater COD was highest when the pH value was 7.50. On the one hand, some studies showed that pH could affect catalytic performance by influencing the active sites of catalysts [35,57]. On the other hand, some other studies that organic pollutants in wastewater exhibited different valence states at different pH values, which may also lead to differences in organic matter removal efficiency [37,58]. This study indicated that under neutral or weakly alkaline conditions, a catalytic oxidation system could produce free radicals more easily and the removal efficiency of organic matter in wastewater was better, which is similar to some previous research results [10,59]. Ozone is a strong oxidant, and increasing its concentration can improve the degradation efficiency of wastewater COD [60]. The results showed that the higher the ozone concentration within a certain range, the better the COD removal effect, which may be due to the gradual increases in the types of oxides in the reaction system as the concentration of ozone increases [61,62]. In addition, the increase in catalyst concentration also promoted the improvement of organic matter removal efficiency. The results may be explained by an increase in the ozone catalyst concentration, causing an increase in the concentration of free radicals in the reaction system, leading to the efficient removal of wastewater COD [63].Notably, when the catalyst dosage was excessively high, the degradation efficiency of the wastewater did not increase significantly, which may have resulted from free-radical quenching [64]. The total organic matter content of the wastewater has a substantial impact on the treatment effect of catalytic ozone oxidation [65]. In addition, in actual wastewater treatment processes, variability in wastewater quality and quantity is relatively strong. In this study, the optimal initial COD concentration was 400 mg/L. When the initial concentration of organic matter in wastewater is excessively low, there may be a degradation limit for the catalytic oxidation of ozone. When the initial organic matter concentration of wastewater is excessively high, the ozone catalytic oxidation process may cause a lower removal efficiency of wastewater COD, owing to insufficient concentrations of oxidizing agents in the reaction system or catalyst deactivation [38].

The degradation kinetics of organic pollutants in wastewater could provide a theoretical guide for efficient degradation of organic matter [66]. In this study, the kinetic analysis indicated the pseudo-second-order reaction kinetic model had the advantage in investigating the degradation process of organic matter, which is similar to the previous research results [38,67]. The kinetic analysis of the catalytic ozonation process for the degradation of organic pollutants has not yet reached a unified conclusion. In previous studies, different research results indicated that the degradation process of organic matter may follow zero order reaction kinetics, pseudo-first-order reaction kinetics, two-stage reaction kinetics, or pseudo-third-order reaction kinetics [30,68,69,70]. The reason for the above phenomenon may be due to differences in reaction systems and water quality among different studies. In this study, considering the complexity of wastewater quality, pseudo-second-order reaction kinetics was more suitable for describing the degradation process of organic pollutants.

### 4.3. Analysis of Reasons for Catalyst Deactivation

By comparing the catalytic performance of the catalyst before and after use, it could be concluded that RO concentrate could reduce the catalytic activity of the catalyst. Notably, the treatment efficacy of the used catalyst on wastewater was higher than that of the single ozone system, indicating that the catalyst had a high stability. Moreover, SEM-EDS analysis showed that the composition in the catalyst surface may be a result of organic pollutants and inorganic salts, such as calcium ions, in the RO concentrate water covering the active components on the catalyst surface. The above results may have occurred due to the deposition of salts and organic pollutants in the RO concentrate water on the catalyst surface. Notably, the loss of the loose structure of the catalyst may lead to surface cementation of the catalyst, which in turn may reduce its catalytic activity [10].The result of BET analysis suggests that the catalyst was contaminated and experienced pore blockage, potentially leading to the inactivation of some catalytic active sites and ultimately affecting the COD removal efficiency in wastewater [24]. In addition, the crystal phases of the used catalysts did not show significant changes, which may have resulted from the pollutants affecting the catalytic activity being non-crystalline substances or present at relatively low concentrations [71]. The result of TG analysis revealed the presence of refractory substances on the surface of the used catalyst, such as calcium oxide and magnesium oxide, which do not easily decompose at high temperatures. XPS results also proved the presence of calcium oxide and magnesium oxide in the used catalyst [47]. XRF results suggest that the used catalyst may contain calcium and magnesium oxides, which may represent the key substances that caused deactivation of the catalyst. Notably, the percentages of calcium oxide, magnesium oxide, chlorine, sulfur, and fluorine contents in the elemental composition of the used catalyst increased significantly. This increase may have resulted from the deposition or adsorption of these inorganic salt ions onto the catalyst surface through a series of physicochemical reactions when the catalyst was used to treat the RO concentrate. Combining the BET analysis results mentioned earlier, the deposition substances formed from the above elements may constitute an important reason for the changes in the catalyst’s average pore diameter, pore volume, specific surface area, etc.

**Figure 12:**
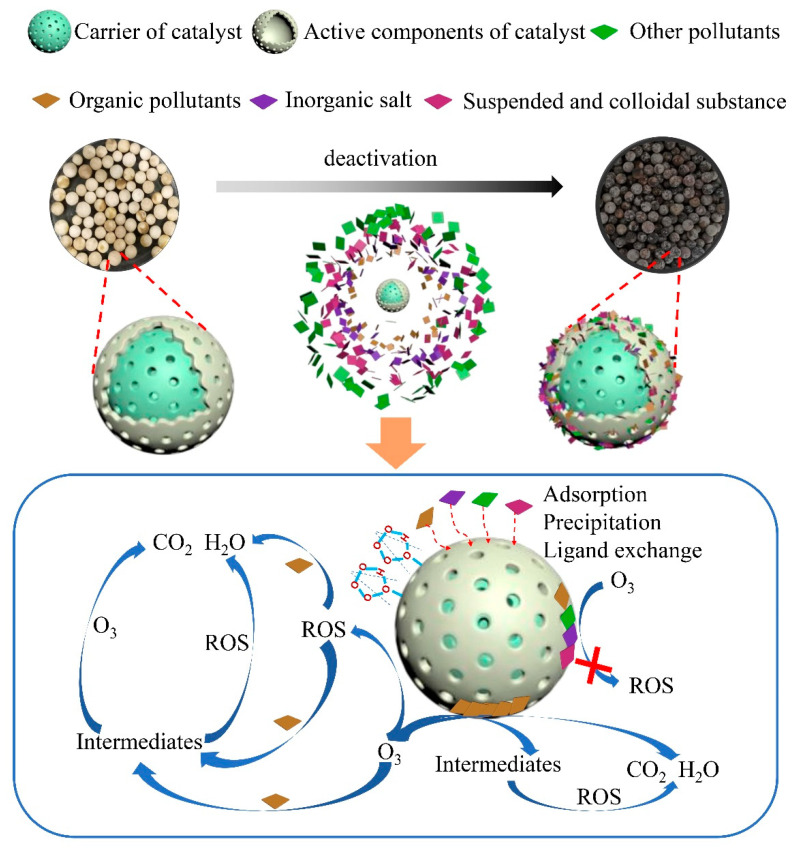
Schematic diagram of possible deactivation mechanism of the catalyst.

At present, there is no consensus on the reasons for catalyst deactivation. It is reported that carbon deposition in the internal pore structure of catalysts is the main cause of catalyst deactivation [22,23]. In addition, some research results also indicated that the loss of catalytic active components is closely related to catalyst deactivation [72]. Meanwhile, some other studies suggest that inorganic salt deposition may be an important cause of catalyst deactivation [10,25]. Considering the possible differences between different studies, the reasons for catalyst deactivation could not be generalized. In this study, in combining the content mentioned previously, and the catalyst has a mesoporous “core-shell” structure, which is supported on aluminum oxide, with manganese oxide and cerium oxide as the main active components. The catalyst deactivation process is illustrated in Figure 12. During the catalytic ozonation process for treating the RO concentrate, the catalyst encountered organic pollutants, inorganic salts, suspended solids, colloids, microorganisms, and their byproducts in the wastewater. The pollutants in wastewater adhered to or were embedded on the surface or inside the catalyst through physical adsorption, chemical adsorption, precipitation, coordination exchange, and other processes. The concentration of reactive oxygen species in the reaction system and the removal efficiency of organic substances from wastewater were reduced through affecting the catalytic activity of the active sites on the catalyst surface. Notably, the deposition of inorganic salts such as calcium and magnesium ions may have represented the main cause of catalyst deactivation.

## 5. Future Research and Perspectives

In subsequent studies, methods to delay catalyst deactivation during actual processes should be further explored. Avenues for future research could include increasing the hardness removal and other pretreatment processes and regularly replacing or regenerating the ozone catalyst during the actual catalytic ozonation process for high-salt wastewater treatment to slow the deactivation process of the catalyst. Moreover, in future research, it is also important to further investigate the changes in other water quality indicators in the wastewater before and after treatment with the catalytic ozonation process. In addition, more comprehensive characterization methods should be used to further explore the specific causes and processes of catalyst deactivation in future research.

## 6. Conclusions

This study utilized a catalytic ozonation process to treat a coal chemical RO concentrate, explored the basic water quality indicators of this concentrate, investigated the removal efficiency and degradation kinetics of COD in wastewater under different parameter conditions, and examined the spectroscopic changes before and after the treatment of the RO concentrate. Furthermore, the possible reasons for the deactivation of the ozone catalyst and its mechanisms were analyzed. The results obtained revealed that the RO concentrate had large water quality fluctuations, high organic pollutant and salt contents, a high hardness, and high suspended solids. The optimal pH, initial pollutant concentration, ozone concentration, and catalyst concentration for the catalytic ozonation process were 7.5, 400 mg/L, 15 mg/L, and 250 g/L, respectively. The results also showed that under the optimal parameters, the removal efficiency of the wastewater COD was 66.75%. Characterization methods such as three-dimensional fluorescence, ultraviolet, and infrared spectroscopy indicated that the organic pollutants in the treated wastewater were largely removed. Analysis methods such as SEM-EDS, BET, XRD, TG, XRF, and XPS, combined with catalytic performance experiments, indicated that the deposition of inorganic salts, such as calcium and magnesium, may be an important explanation of the deactivation of the catalyst. In such actual wastewater treatment engineering, the service life of the catalyst can be prolonged through additional hardness removal pretreatment processes (or catalyst regeneration), thereby ensuring the operational stability of the catalytic ozonation process.

## Figures and Tables

**Table 1:** Water quality indicators of coal chemical RO concentrate.

Water Quality Indicators	Numerical Value
COD (mg/L)	200–600
pH	6.5–9
Electrical conductivity (μs/cm)	29,000–33,000
SO_4_^2−^ (mg/L)	1300–3100
Cl^−^ (mg/L)	12,000–16,000
Alkalinity (mg/L)	3000–6000
Br^−^ (mg/L)	880–1200
NO_2_^−^ (mg/L)	900–1300
F^−^ (mg/L)	15–25
Calcium hardness (mg/L)	100–330
Total hardness (mg/L)	300–1000
Turbidity (NTU)	16–77
Suspended solids (mg/L)	32–133
Color (°)	1985.4–2183.6

**Table 2:** λ and R^2^ values from COD degradation in wastewater at different pH values.

pH	Pseudo-First-Order Kinetic		Pseudo-Second-Order Kinetic	
	λ_1_	R^2^	λ_2_ (×10^−5^)	R^2^
	ln (*C_t_*/*C*_0_) − *T*		1/*C_t_* − *T*	
4.5	−0.0176	0.9905	6.87	0.9940
6	−0.0193	0.9841	7.80	0.9890
7.5	−0.0205	0.9863	8.55	0.9941
9	−0.0185	0.9847	7.34	0.986
10.5	−0.0152	0.9934	5.55	0.9950

**Table 3:** λ and R^2^ values from COD degradation in wastewater at different catalyst concentrations.

Catalyst Concentration (g/L)	Pseudo-First-Order Kinetic		Pseudo-Second-Order Kinetic	
	λ_1_	R^2^	λ_2_ (×10^−5^)	R^2^
	ln (*C_t_*/*C*_0_) − *T*		1/*C_t_* − *T*	
100	−0.0156	0.9877	5.75	0.9890
150	−0.0174	0.9869	6.72	0.9900
200	−0.0190	0.9843	7.65	0.9850
250	−0.0205	0.9863	8.55	0.9940
300	−0.0216	0.9787	9.27	0.9820

**Table 4:** λ and R^2^ values from COD degradation in wastewater at different ozone concentrations.

Ozone Concentration (mg/L)	Pseudo-First-Order Kinetic		Pseudo-Second-Order Kinetic	
	λ_1_	R^2^	λ_2_ (×10^−5^)	R^2^
	ln (*C_t_*/*C*_0_) − *T*		1/*C_t_* − *T*	
5	−0.0118	0.9973	3.97	0.9970
10	−0.0169	0.9917	6.45	0.9960
15	−0.0205	0.9863	8.55	0.9940
20	−0.0217	0.9815	9.36	0.9840
25	−0.0241	0.9739	11.1	0.9600

**Table 5:** Pseudo-second-order kinetic equations (1/*C_t_* − *T*) for COD degradation in wastewater under different parameters.

Parameter Name	Parameter Value	Kinetic Equation
pH	4.5	y = 0.0025 + 6.87 × 10^−5^ x
	6	y = 0.0025 + 7.80 × 10^−5^ x
	7.5	y = 0.0025 + 8.55 × 10^−5^ x
	9	y = 0.0025 + 7.34 × 10^−5^ x
	10.5	y = 0.0025 + 5.55 × 10^−5^ x
Catalyst concentration	100 g/L	y = 0.0025 + 5.75 × 10^−5^ x
	150 g/L	y = 0.0025 + 6.72 × 10^−5^ x
	200 g/L	y = 0.0025 + 7.65 × 10^−5^ x
	250 g/L	y = 0.0025 + 8.55 × 10^−5^ x
	300 g/L	y = 0.0025 + 9.27 × 10^−5^ x
Ozone concentration	5 mg/L	y = 0.0025 + 3.97 × 10^−5^ x
	10 mg/L	y = 0.0025 + 6.45 × 10^−5^ x
	15 mg/L	y = 0.0025 + 8.55 × 10^−5^ x
	20 mg/L	y = 0.0025 + 9.36 × 10^−5^ x
	25 mg/L	y = 0.0025 + 1.11 × 10^−4^ x

**Table 6:** Relative percentage of elemental analysis before and after catalyst usage.

Element	Fresh Catalyst		Used Catalyst	
	Weight Percentage (%)	Atomic Percentage (%)	Weight Percentage (%)	Atomic Percentage (%)
C	13.51	20.95	12.49	17.94
O	41.49	48.30	59.78	64.46
Mg	0.00	0.00	0.00	0.00
Al	44.31	30.59	27.13	17.35
Ca	0.00	0.00	0.61	0.26
Mn	0.30	0.10	0.00	0.00
Ce	0.39	0.05	0.00	0.00

**Table 7:** XRF spectrum analysis of the catalyst before and after use.

Percentage Content (%)	Fresh Catalyst	Used Catalyst
Al_2_O_3_	89.33	84.4
SiO_2_	7.6	7.46
MnO	1.14	0.99
CeO_2_	0.742	0.634
Na_2_O	0.495	1.55
K_2_O	0.17	0.144
MgO	0.145	2.29
CaO	0.132	1.09
Cl	0.0542	0.665
SO_3_	0.0369	0.336
F	Not detected	0.218

## Data Availability

The original data presented in this study are included in the article. Further inquiries can be directed to the corresponding author.

## Supplementary Materials

The following supporting information can be downloaded at: https://www.mdpi.com/article/10.3390/toxics12090681/s1, Figure S1: Thermogravimetric curves of the a. fresh catalyst and the b. used catalyst; Figure S2: XPS spectral analysis of the catalyst a. before and b. after use.
