# Supplementary material for: Catalytic Ozonation Treatment of Coal Chemical Reverse Osmosis Concentrate: Water Quality Analysis, Parameter Optimization, and Catalyst Deactivation Investigation
Source: Toxics. 2024 Sep 20;12(9):681. doi: 10.3390/toxics12090681 (PMC11435963; doi:10.3390/toxics12090681)
Supplement: Supplementary file 1 [file toxics-12-00681-s001.zip › toxics-3180787-supplementary.pdf]

Figure S1

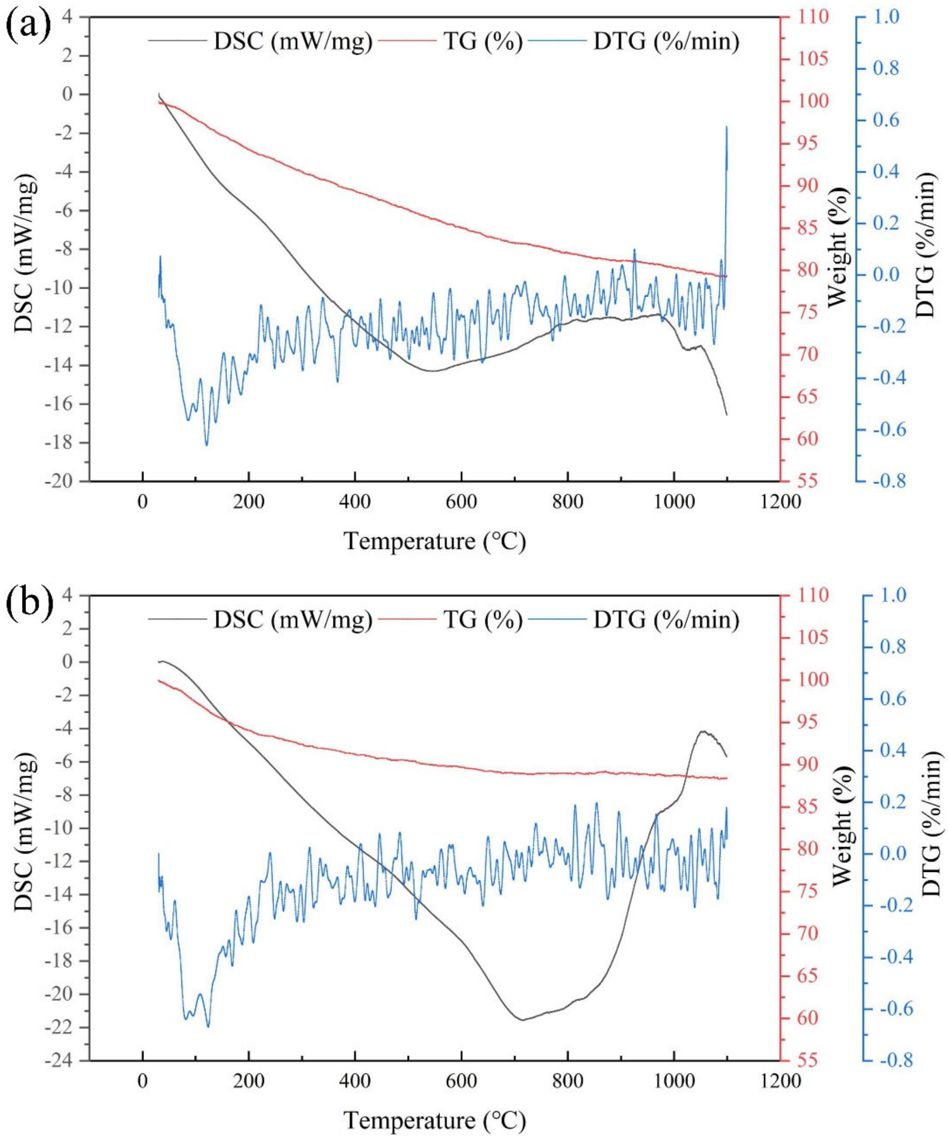

Figure S1. Thermogravimetric curves of the a. fresh catalyst and the b. used catalyst.

Figure S2

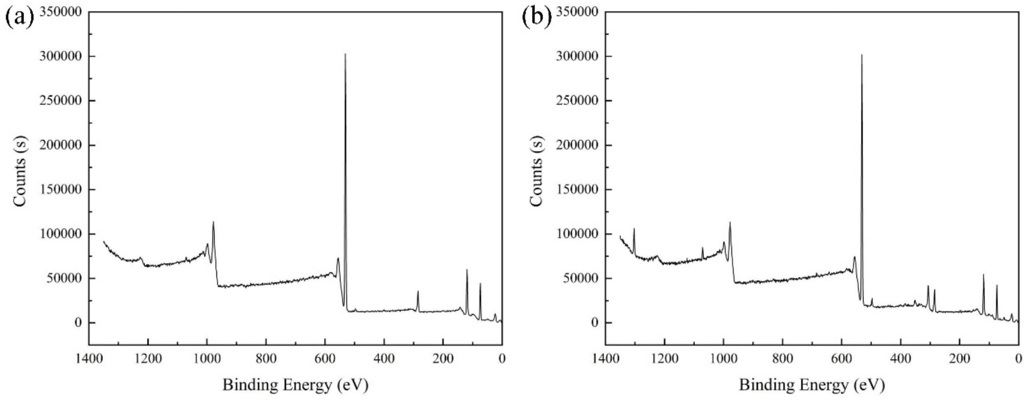

Figure S2. XPS spectral analysis of the catalyst a. before and b. after use.
